# Transcriptional Co‐Activator With PDZ Binding Motif (TAZ) Inhibits Dexamethasone‐Induced Muscle Atrophy via mTOR Signalling

**DOI:** 10.1002/jcsm.13790

**Published:** 2025-03-30

**Authors:** Kyung Min Kim, Ho Taek Oh, Youjin Do, Gi Don Yoo, Woong Heo, Jeekeon Park, Hyejin Yang, Suh Jin Yoon, Mi Ran Byun, Eun Sook Hwang, Jeong‐Ho Hong

**Affiliations:** ^1^ Division of Life Sciences Korea University Seoul South Korea; ^2^ College of Pharmacy and Graduate School of Pharmaceutical Sciences Ewha Woman's University Seoul South Korea; ^3^ College of Pharmacy Daegu Catholic University Gyeongsan South Korea; ^4^ Program in Innovative Biomaterials Convergence Ewha Womans University Seoul South Korea

**Keywords:** dexamethasone, ginsenoside, mTOR, muscle atrophy

## Abstract

**Background:**

Glucocorticoid therapy has a beneficial effect in several diseases, but chronic treatment has adverse effects, including muscle atrophy, which refers to the gradual decrease in muscle mass, size and strength. It is important to know how the muscle atrophy occurs, but the underlying mechanism is not yet fully understood. This study shows that dexamethasone decreases levels of the transcriptional co‐activator with PDZ binding motif (TAZ), which facilitates dexamethasone‐induced muscle atrophy.

**Methods:**

To induce muscle atrophy, C2C12 myotubes were treated with dexamethasone, and mice were fed with water containing dexamethasone. Muscle atrophy was analysed for the expression of myosin heavy chain, MuRF1 and Atrogin‐1 using immunofluorescence staining, immunoblot analysis and qRT‐PCR. Muscle tissue was analysed by haematoxylin and eosin staining. Adeno‐associated virus was used for overexpression of wild‐type and mutant TAZ.

**Results:**

TAZ levels decrease in dexamethasone‐treated mice (0.36‐fold, *p* < 0.001) and C2C12 myotubes (0.44‐fold, *p* = 0.024). Overexpression of the TAZ mutant, which resists its proteolytic degradation, inhibits dexamethasone‐induced muscle atrophy. Atrogin‐1 and MuRF1 interact with TAZ and facilitate its degradation in dexamethasone‐treated C2C12 myotubes. TAZ mutant stimulates protein synthesis through activation of mTOR signalling via induction of RhebL1 (DEX; Con vs, TAZ4SA: 5.1‐fold, *p* < 0.001) in dexamethasone‐treated mice. Ginsenoside Rb3 increases TAZ levels in dexamethasone‐treated mice (1.49‐fold, *p* = 0.007) and C2C12 myotubes (1.63‐fold, *p* = 0.01), which stimulates mTOR signalling and inhibits dexamethasone‐induced muscle atrophy.

**Conclusions:**

Our results demonstrate a novel regulatory mechanism of dexamethasone‐induced muscle atrophy by TAZ, suggesting that stabilisation of TAZ in muscle cells ameliorates the muscle atrophy. These results suggest that TAZ may be a drug target for the dexamethasone‐induced muscle atrophy.

## 1 Introduction

Muscle atrophy refers to the wasting or shrinking of muscle tissue. It is caused by several factors including lack of physical activity, immobilisation due to injury and chronic glucocorticoid treatment [1]. Dexamethasone has been used clinically to inhibit inflammation in several conditions, but long‐term treatment leads to adverse effects, including muscle atrophy. Dexamethasone induces the Atrogin‐1 and Murf1 [2, 3, 4], which are involved in the ubiquitin‐proteasome pathway, and leads to a decrease in muscle proteins [5, 6]. In addition, dexamethasone inhibits the mTOR signalling [4, 7], which facilitates protein synthesis in muscle cells, and the anabolic pathway driven by IGF‐1 [8, 9]. Several strategies, including exercise, can be used to alleviate dexamethasone‐induced muscle atrophy, but better therapies are needed to manage this adverse effect.

Transcriptional co‐regulators, transcriptional co‐activators with PDZ binding motif (TAZ) and Yes‐associated protein (YAP) interact with multiple transcription factors to regulate tissue homeostasis and regeneration. Various extracellular signals, including Hippo, Wnt and GPCR, and mechanical signals regulate TAZ/YAP activity [10, 11, 12, 13, 14]. The signals regulate the stability of TAZ/YAP and their nuclear localisation. In the Hippo signalling pathway, Lats kinases phosphorylate TAZ/YAP, which induces their proteolytic degradation and cytosolic localisation by binding to 14‐3‐3 proteins [15, 16, 17, 18]. Inactivation of Hippo signalling stabilises TAZ/YAP, facilitating its nuclear localisation and interaction with several transcription factors, including members of the transcriptional enhancer factor TEF family (TEADs) [19, 20]. TAZ has been reported to stimulate myogenic differentiation and muscle regeneration [21, 22]. TAZ also stimulates mitochondrial biogenesis and muscle adaptation via mTOR signalling [23]. Recently, TAZ activated muscle satellite cells to stimulate muscle regeneration [24]. However, the effect of TAZ has not been investigated in pathological conditions such as dexamethasone‐induced muscle atrophy.

Here, we investigated the protective role of TAZ in dexamethasone‐induced muscle atrophy.

## 2 Materials and Methods

### 2.1 Mice

Seven‐week‐old male C57BL/6J mice were housed in a sterile facility at 22°C with a 12‐h light/dark cycle and 50% humidity, with free access to water and standard rodent chow. All animal care and experimental procedures were approved by the Animal Care and Use Committee of Korea University (KUIACUC‐2019‐0093) and followed institutional guidelines.

### 2.2 Cell Culture

C2C12 myoblasts (ATCC, CRL‐1772) were maintained at 37 °C in a 5% CO_2_ atmosphere in DMEM supplemented with 20% fetal bovine serum, 100 units/mL penicillin and 100 μg/mL streptomycin. C2C12 TAZ and TAZ4SA cell lines were generated by retroviral transduction. Phoenix cells were transfected with the respective retroviral vectors (pBabe‐TAZ or pBabe‐TAZ4SA plasmids) together with viral packaging vectors. After 24 h of transfection, C2C12 cells were infected with the virus‐containing medium supplemented with 4 μg/mL polybrene. Transfected cells were selected by culturing them in growth medium containing 2 μg/mL puromycin. To induce differentiation into myotubes, C2C12 myoblasts were cultured at 80%–90% confluence in DMEM containing 2% horse serum.

### 2.3 Dexamethasone‐Induced Muscle Atrophy

For in vitro atrophy induction, C2C12 cells were differentiated into myotubes and then treated with 10 μM dexamethasone for 24 h. For in vivo atrophy induction, 7‐week‐old male mice were given approximately 1 mg/kg/day of water‐soluble dexamethasone in their drinking water for 10 days [25].

### 2.4 Ginsenoside Treatment

For in vitro studies, after inducing atrophy in myotubes with dexamethasone, the myotubes were treated with 5 μM ginsenosides Rb3, Rg1 and Rb1 for 2 days. For in vivo studies, ginsenoside Rb3 was administered orally at a dose of 10 mg/kg during dexamethasone‐induced muscle atrophy [26].

### 2.5 Immunoblot Analysis

Cell lysates were prepared using TNE lysis buffer (20 mM Tris–HCl [pH 7.5], 150 mM NaCl, 2 mM EDTA, 1% NP‐40, 50 mM NaF and 1 mM Na₃VO₄) containing protease inhibitors. For tissue lysates, dissected tissues were homogenised in pre‐chilled RIPA buffer (50 mM Tris–HCl [pH 7.4], 150 mM NaCl, 1 mM EDTA, 1% NP‐40, 0.5% sodium deoxycholate, 0.1% SDS, 1 mM NaF and 1 mM Na₃VO₄) containing protease inhibitors using a tissue grinder. The lysates were denatured in SDS sample buffer, resolved by SDS‐PAGE and transferred to PVDF membranes. The membranes were blocked with 5% non‐fat dry milk in Tris‐buffered saline with Tween 20 (TBST) and incubated overnight at 4°C with primary antibodies against different proteins. The membranes were then incubated with HRP‐conjugated secondary antibodies for 1 h, washed three times with TBST for 5 min each and visualised using the enhanced chemiluminescence (ECL) system. Antibodies against TAZ/YAP (8418), Rheb (13879), Vinculin (13901), p‐p70 S6K (9234) and p70 S6K (2708) were purchased from Cell Signaling Technology. Myosin heavy chain antibody (MF20) was obtained from the Developmental Studies Hybridoma Bank. Anti‐Atrogin‐1 (AP2041) and MuRF1 (MP3401) antibodies were purchased from ECM Biosciences. Anti‐α‐tubulin (sc‐5286) and RhebL1 (sc‐514 095) antibodies were purchased from Santa Cruz Biotechnology.

### 2.6 Quantitative Real‐Time PCR Analysis

Total RNA was isolated from cells and tissues using TRIzol and reverse transcribed into cDNA using M‐MLV reverse transcriptase. Real‐time PCR was performed on a LightCycler 480 system (Roche). Relative transcript levels were calculated by normalising the cycle threshold (Ct) values to that of GAPDH. Primers used to analyse target gene expression are listed in Table 1.

**TABLE 1:** Primers for qRT‐PCR analysis.

Gene	Forward	Reverse
*Gapdh*	5′‐GCTTGTCATCAACGGGAAG‐3′	5′‐GATGTTAGTGGGGTCTCG‐3′
*Taz*	5′‐GTCACCAACAGTAGCTCAGATC‐3′	5′‐GGACACTGTAGCACCCTAACCCCA‐3′
*Atrogin‐1*	5′‐CTCTGTACCATGCCGTTCCT‐3′	5′‐GGCTGCTGAACAGATTCTCC‐3′
*MuRF1*	5′‐TGTCTGGAGGTCGTTTCCG‐3′	5′‐TGCCGGTCCATGATCACTT‐3′

### 2.7 Immunofluorescence Staining

Myotubes were fixed in 4% paraformaldehyde for 15 min at room temperature. After blocking with PBS containing 0.3% Triton X‐100 and 5% BSA, samples were incubated with myosin heavy chain antibody (DSHB, MF20) at 4°C overnight, followed by incubation with fluorescent dye‐conjugated secondary antibodies for 2 h. Cell nuclei were counterstained with DAPI (Vector Laboratories, H‐1500), and fluorescence was observed using a confocal microscope (Carl Zeiss LSM 700) or a fluorescence microscope (Axio Observer 7, Carl Zeiss) at the Ewha Drug Development Core Center.

### 2.8 H&E Staining

Gastrocnemius (GA) muscles were isolated from mice, embedded in paraffin and sectioned. Sections were deparaffinised, rehydrated and stained with Harris haematoxylin solution (Sigma, HHS16), followed by differentiation in 1% acidic alcohol. Sections were stained with 0.2% ammonia solution and counterstained with eosin Y solution (Sigma, HT110116). Images were captured using a digital camera (Canon, EOS 650D) attached to an upright microscope (Nikon, ECLIPSE Ni).

### 2.9 Immunoprecipitation Analysis

FLAG‐TAZ‐ or FLAG‐TAZ4SA‐overexpressing C2C12 cells were differentiated into myotubes, followed by treatment with MG132 in combination with either vehicle or dexamethasone. After treatment, cells were lysed in RIPA buffer [150 mM NaCl, 50 mM Tris–HCl (pH 7.4), 1 mM EDTA, 1% NP‐40, 0.5% sodium deoxycholate, 0.1% SDS, 1 mM Na₃VO₄ and 1 mM NaF] containing protease inhibitors. Lysates were pre‐cleared with BSA‐coated Sepharose beads (Sigma‐Aldrich, CL4B200) and then incubated with anti‐FLAG M2 affinity gel (Sigma‐Aldrich, A2220) for 2 h at 4°C. After washing with lysis buffer, the bound proteins were denatured and analysed by immunoblotting.

### 2.10 Generation of Atrogin‐1 or MuRF1 Knockout Cell Lines

pSpCas9‐2A‐Puro (PX459) v2.0 was a gift from Feng Zhang (Addgene plasmid #62988) [27]. For Atrogin‐1 or MuRF1 knockout (KO) plasmids, the synthesised DNA oligomers were annealed and amplified. The amplified DNAs were ligated into PX459 using Gibson assembly. The sequences of the oligomers are listed in Table 2. C2C12 cells were transfected with the plasmids using the NEPA 21 electroporator. Stable cell lines were selected in puromycin to generate Atrogin‐1 and MuRF1 KO cell lines. KO efficiency was determined by immunoblot analysis.

**TABLE 2:** Primers for knockout plasmids.

Gene	Forward	Reverse
*Atrogin‐1*	5′‐ATCTTGTGGAAAGGACGAAA**AAGTGGATCTATGTTCACAA**‐3′	5′‐GCTATTTCTAGCTCTAAAAC**TTGTGAACATAGATCCACTT**‐3′
*Murf1*	5′‐ATCTTGTGGAAAGGACGAAA**AAGTGATCATGGACCGGCAC**‐3′	5′‐GCTATTTCTAGCTCTAAAAC**GTGCCGGTCCATGATCACTT**‐3′

### 2.11 Adeno‐Associated Virus Production and Local Transduction

The adeno‐associated virus (AAV) vectors for overexpressing TAZ or TAZ4SA were constructed using pBabe‐TAZ or pBabe‐TAZ4SA plasmids. The TAZ or TAZ4SA coding sequences were subcloned into the multiple cloning site of the pAAV6‐CMV plasmid (Takara Bio, Cat. No. 6665), which served as the template for AAV production. AAV particles for TAZ or TAZ4SA overexpression were generated using the AAVpro Helper Free System (Takara Bio, Cat. No. 6651) according to the manufacturer's protocol. Briefly, AAVpro 293T cells (Takara Bio, Cat. No. 632273) were seeded on 150 mm dishes and transfected with the AAV plasmid constructs using TransIT‐VirusGEN Transfection Reagent (Mirus Bio, MIR 6700). AAV particles were isolated from the cell pellets using AAV Extraction Solution (Takara Bio, Cat. No. 6666), and the viral titre was determined using the AAVpro Titration Kit (Takara Bio, Cat. No. 6233). For local transduction in the gastrocnemius muscle, mice were anaesthetised with isoflurane, and 50 μL of AAV containing 5 × 10^10^ vector genomes was injected into the GA muscle. Injections were made at 4 points in the muscle tissue. AAV6‐CMV was used as a control. Two weeks after virus administration, atrophy experiments were performed.

### 2.12 Quantification and Statistical Analysis

For in vitro experiments, all data are presented as the mean ± standard deviation (SD) of at least three independent experiments. For in vivo experiments, all data are presented as the mean ± standard error of the mean (SEM) based on the indicated sample size. Statistical significance was assessed using the appropriate tests: one‐tailed and two‐tailed *t*‐test, one‐way analysis of variance (ANOVA) followed by Tukey's post hoc test, two‐way ANOVA followed by Holm–Sidak's multiple comparisons test, and two‐way repeated measures ANOVA followed by Tukey's post hoc test. Exact *p* values are provided in the graph, and *non‐significant* results are not explicitly indicated. A *p* value of less than 0.05 was considered statistically significant, whereas a *p* value greater than 0.05 was considered *non‐significant*. GraphPad Prism 6 (v6.01) was used for statistical analysis and graph preparation.

## 3 Results

### 3.1 Dexamethasone Reduces TAZ Levels in Muscle

**FIGURE 1:**
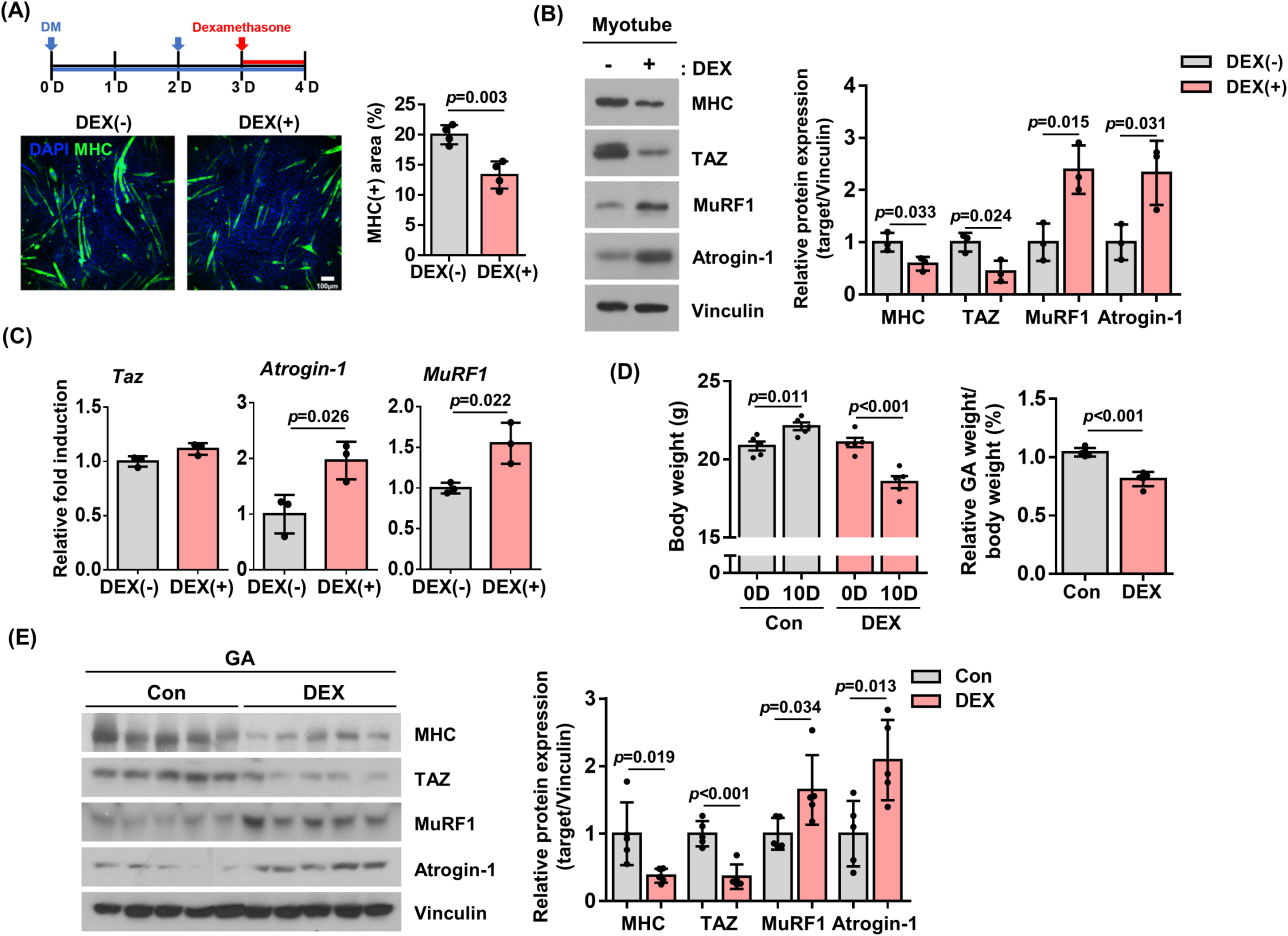
Dexamethasone reduces TAZ levels in skeletal muscle. (A) Schematic representation of the experimental design for dexamethasone treatment in C2C12 myotubes. DM refers to differentiation media. Bottom panel shows the levels of myosin heavy chain (MHC) by immunofluorescence staining in vehicle‐treated [DEX(−)] and dexamethasone [DEX(+)]‐treated myotubes. Scale bar, 100 μm. The area of MHC was quantified from the immunofluorescence staining (*n* = 3). (B) Left: The levels of MHC, TAZ, MuRF1, Atrogin‐1, and vinculin in Panel (A) were analysed by immunoblotting. Vinculin was used as a loading control. Right: The levels of MHC, TAZ, MuRF1, and Atrogin‐1 were quantified (*n* = 3). (C) The transcript levels of *Taz*, *Atrogin‐1* and *MuRF1* were analysed by quantitative reverse transcription polymerase chain reaction (qRT‐PCR) in Panel (A) (*n* = 3). (D) Left: Mice were administered drinking water containing dexamethasone, and body weights were analysed at the indicated time points (*n* = 5). Right panel depicts the ratio of gastrocnemius (GA) muscle weight to total body weight in the left panel. (E) The levels of MHC, TAZ, MuRF1, Atrogin‐1, and vinculin in Panel (D) were analysed by immunoblotting. Vinculin was used as a loading control. Right: The levels of MHC, TAZ, MuRF1 and Atrogin‐1 were quantified (*n* = 5). The data are presented as the mean ± SD for (A), (B) and (C) and as the mean ± SEM for (D) and (E). Statistical analysis for all figures was performed using two‐tailed *t*‐test. Exact *p* values are provided in the graph, and *non‐significant* results are not explicitly indicated.

TAZ has been demonstrated to stimulate muscle regeneration following both muscle injury and exercise [21, 24]. A further investigation was conducted to elucidate the role of TAZ in dexamethasone‐induced muscle atrophy. C2C12 myoblast cells were differentiated into myotubes and subsequently treated with dexamethasone. As shown in Figure 1A, dexamethasone was found to diminish the levels of myosin heavy chain (MHC), a marker of muscle differentiation in a fluorescence immunostaining experiment (0.62‐fold, *p* = 0.003). The decreased MHC levels were further confirmed by immunoblot analysis (0.59‐fold, *p* = 0.033) (Figure 1B). Additionally, the levels of MuRF1 and Atrogin‐1, which are markers of muscle atrophy, were observed to have increased in dexamethasone‐treated cells (MuRF1: 2.39‐fold, *p* = 0.015, Atrogin‐1: 2.33‐fold, *p* = 0.031). Notably, the levels of TAZ were found to be reduced following dexamethasone treatment (0.44‐fold, *p* = 0.024) (Figure 1B). However, the expression of TAZ transcripts was not altered (1.12‐fold, *non‐significant*), whereas that of Atrogin‐1 and MuRF1 was increased following dexamethasone treatment (*Atrogin‐1*: 1.96‐fold, *p* = 0.026, *MuRF1*: 1.55‐fold, *p* = 0.022) (Figure 1C). The results indicate that dexamethasone promotes the proteolytic degradation of TAZ in myotubes. Subsequently, the in vivo effect of dexamethasone on TAZ levels was investigated by administering dexamethasone to mice and analysing their muscle weight. As shown in Figure 1D, the administration of dexamethasone resulted in a reduction in both total body weight (DEX; 0D vs. 10D: 0.87‐fold, *p* < 0.001) and gastrocnemius muscle weight relative to body weight (0.78‐fold, *p* < 0.001). Additionally, TAZ levels in muscle were found to be reduced in conjunction with decreased MHC levels (TAZ: 0.36‐fold, *p* < 0.001, MHC: 0.38‐fold, *p* = 0.019) following dexamethasone administration (Figure 1E). These results collectively indicate that TAZ may serve as an effector of dexamethasone‐induced muscle atrophy.

### 3.2 TAZ Mutant Inhibits Dexamethasone‐Induced Muscle Atrophy

**FIGURE 2:**
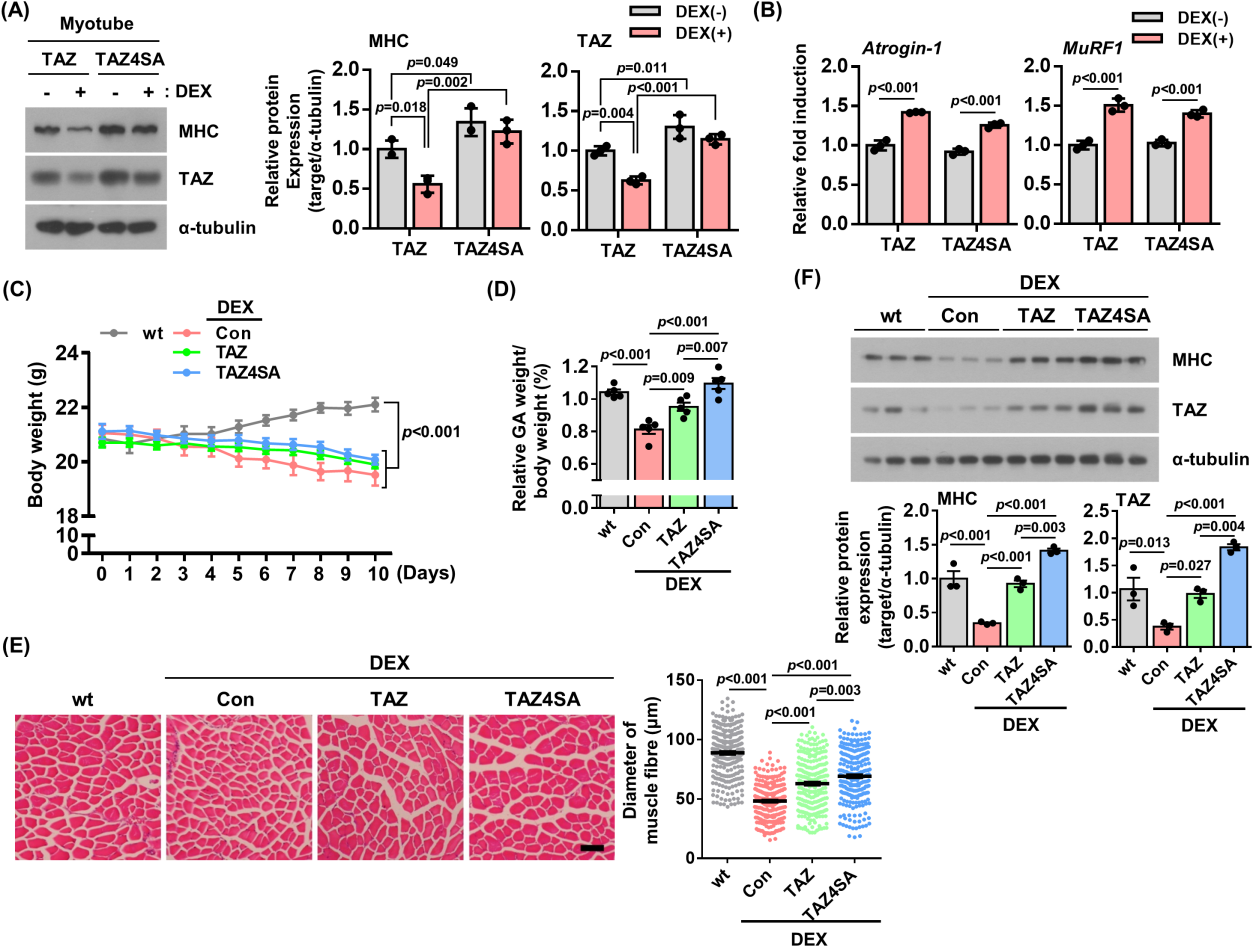
TAZ mutant inhibits dexamethasone‐induced muscle atrophy. (A) TAZ wild‐type (TAZ) or mutant (TAZ4SA) overexpressing C2C12 myotubes were treated with either a vehicle [DEX(−)] or dexamethasone [DEX(+)]. The levels of MHC, TAZ and α‐tubulin were analysed by immunoblotting. α‐Tubulin was used as a loading control. Right: The levels of MHC and TAZ were quantified (*n* = 3) (B) The transcript levels of *Atrogin‐1* and *MuRF1* were analysed by qRT‐PCR in Panel (A) (*n* = 3). (C) Control (Con), TAZ or TAZ4SA AAV viruses were introduced into the GA muscle of mice. Following the administration of dexamethasone, the body weights of the subjects were analysed at the indicated time points (*n* = 5). (D) The ratio of GA muscle weight to total body weight was analysed in Panel (C). (E) Left: Histological examination of the GA muscles using haematoxylin and eosin (H&E) staining in panel (C). Scale bar, 50 μm. Right: Muscle fibre diameters were quantified using the ImageJ software. Each dot on the graph represents the diameter of an individual muscle fibre, and the total number of fibres measured was over 250 per group. Histological analysis was performed on muscle samples from five mice per group. (F) The levels of MHC, TAZ and α‐tubulin in Panel (C) were analysed by immunoblotting. α‐tubulin was used as a loading control. Bottom: The levels of MHC and TAZ were quantified (*n* = 3). The data are presented as the mean ± SD for (A) and (B) and as the mean ± SEM for (C), (D), (E) and (F). Statistical analysis was performed using appropriate tests: Panels (A) and (B) were analysed using two‐way ANOVA followed by Holm–Sidak's multiple comparisons test. Panel (C) was analysed using two‐way repeated measures ANOVA followed by Tukey's post hoc test for multiple comparisons. Panels (D) and (E) were analysed using one‐way ANOVA followed by Tukey's multiple comparisons test. Exact *p* values are provided in the graph, and *non‐significant* results are not explicitly indicated.

The stability of the TAZ protein is regulated by the Hippo signal, and the phosphorylation of TAZ by Lats kinase has been shown to trigger its proteolytic degradation via the ubiquitin–proteasome pathway [16, 17]. We therefore sought to determine whether the phosphorylation‐defective TAZ mutant (TAZ4SA), which has been shown to escape proteasomal degradation, could inhibit dexamethasone‐induced muscle atrophy. TAZ‐ and TAZ4SA‐overexpressing C2C12 cell lines were established and differentiated, and the myotubes were treated with dexamethasone. As shown in Figure 2A, TAZ4SA shows resistance to proteolytic degradation induced by dexamethasone, maintaining MHC levels [TAZ4SA: MHC (0.91‐fold, *non‐significant*), TAZ (0.88‐fold, *non‐significant*)]. Moreover, TAZ4SA exhibited increased MHC and TAZ levels compared to wild‐type TAZ‐overexpressing cells [TAZ vs. TAZ4SA: MHC (1.34‐fold, *p* = 0.049), TAZ (1.3‐fold, *p* = 0.011)]. However, in cells overexpressing wild‐type TAZ, there was a reduction in TAZ and MHC levels following dexamethasone treatment. This was evidenced by a 44% reduction in MHC levels (*p* = 0.018) and a 37% reduction in TAZ levels (*p* = 0.004) (*Figure* 2A). The expression of Atrogin‐1 and MuRF1 transcripts was not altered in cells overexpressing TAZ or TAZ4SA mutants [TAZ: *Atrogin‐1* (1.42‐fold, *p* < 0.001), *MuRF1* (1.51‐fold, *p* < 0.001), TAZ4SA: *Atrogin‐1* (1.37‐fold, *p* < 0.001), *MuRF1* (1.36‐fold, *p* < 0.001)] (*Figure* 2B). To investigate the in vivo effect of TAZ mutants following dexamethasone administration, AAV‐overexpressing TAZ or TAZ4SA mutants were prepared and injected into mouse muscles. Following dexamethasone administration, the mice were subjected to a muscle weight analysis. As shown in Figure 2C, dexamethasone administration resulted in a significant reduction in total body weight [wt vs. DEX; Con (0.86‐fold), TAZ (0.89‐fold), TAZ4SA (0.91‐fold), *p* < 0.001]. However, when the gastrocnemius muscle weight was analysed in relation to the body weight, it was observed that the muscle‐overexpressing TAZ4SA was able to inhibit the decrease in muscle mass (DEX; Con vs. TAZ4SA: 1.35‐fold, *p* < 0.001) (Figure 2D). An increase in the levels of both MHC and TAZ was observed in the TAZ4SA muscle tissue when compared to dexamethasone‐treated control muscle [DEX; Con vs. TAZ4SA: MHC (4.09‐fold, *p* < 0.001), TAZ (4.92‐fold, *p* < 0.001)] (Figure 2F). Furthermore, muscle fibre diameter was also preserved in the TAZ4SA overexpressing muscle (DEX; Con vs. TAZ4SA: 1.45‐fold, *p* < 0.001) (Figure 2E). The results indicate that the TAZ mutant prevents dexamethasone‐induced muscle mass loss, suggesting that TAZ degradation plays a key role in dexamethasone‐induced muscle atrophy.

### 3.3 TAZ Interacts With Atrogin‐1 and MuRF1 for Its Degradation

**FIGURE 3:**
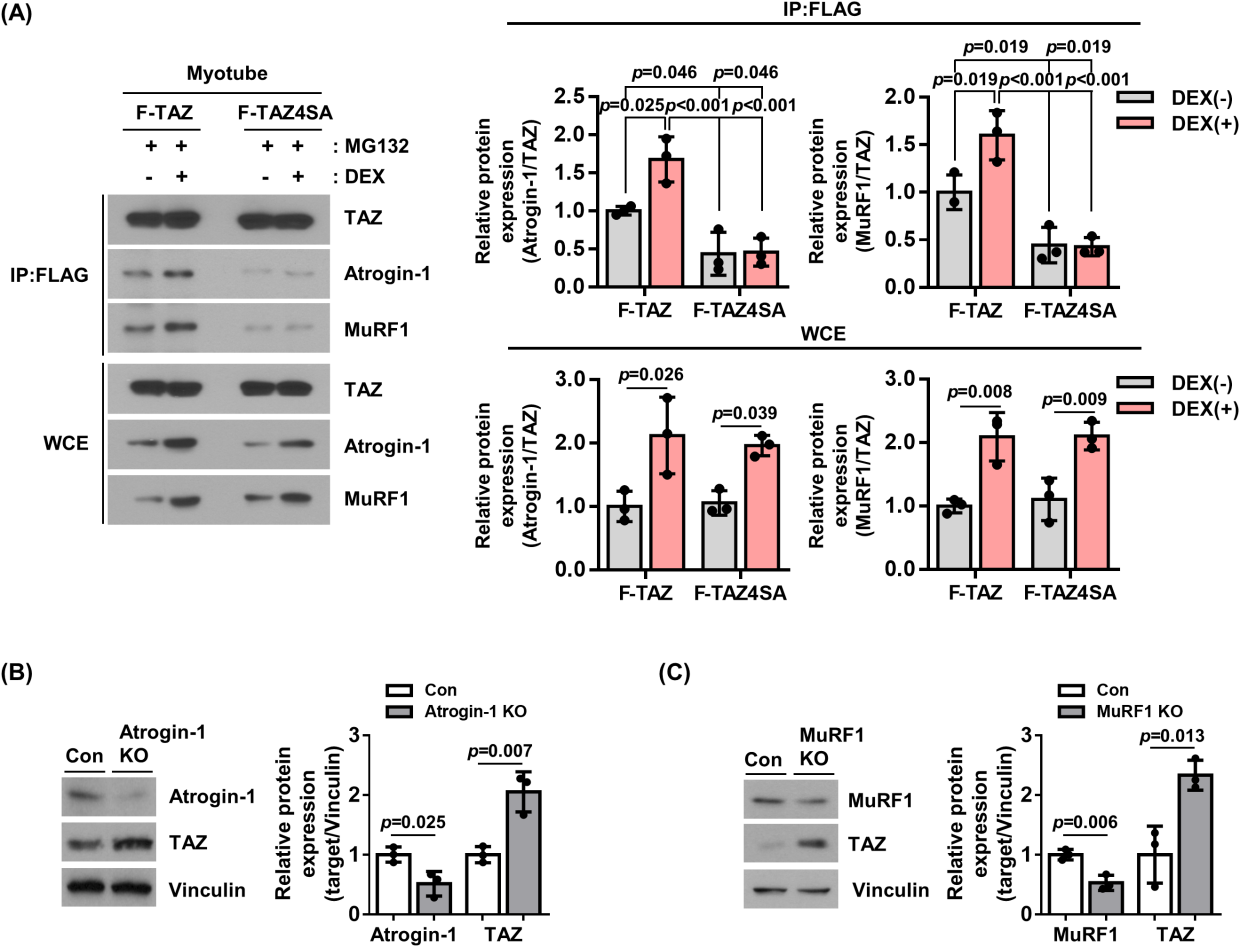
Atrogin‐1 and MuRF1 interacts with TAZ and induces its degradation. (A) Flag‐tagged TAZ (F‐TAZ) and TAZ4SA (F‐TAZ4SA) overexpressing C2C12 myotubes were treated with either a vehicle [DEX(−)] or dexamethasone [DEX(+)] in the presence of MG132. The cell lysates were subjected to immunoprecipitation with anti‐FLAG antibody, and the immune complexes were analysed by immunoblotting for TAZ, Atrogin‐1 and MuRF1. The right panel shows the quantification of the data presented in the left panel (*n* = 3). (B) The CRISPR/Cas9 system was employed to deplete endogenous Atrogin‐1. In Atrogin‐1‐depleted C2C12 myotubes (Atrogin‐1 KO), dexamethasone‐induced TAZ degradation was examined by immunoblotting. The right panel shows the quantification of the data in the left panel (*n* = 3). (C) The CRISPR/Cas9 system was employed to deplete endogenous MuRF1. In MuRF1‐depleted C2C12 myotubes (MuRF1 KO), dexamethasone‐induced TAZ degradation was examined by immunoblotting. The right panel shows the quantification of the data in the left panel (*n* = 3). All data are presented as the mean ± SD. Statistical analysis was performed using appropriate tests: Panel (A) was analysed using two‐way ANOVA followed by Holm–Sidak's multiple comparisons test, and Panels (B) and (C) were analysed using two‐tailed *t*‐test. Exact *p* values are provided in the graph, and *non‐significant* results are not explicitly indicated.

We next investigated whether TAZ engages in a direct physical interaction with Atrogin‐1 and MuRF1. In cells overexpressing Flag‐tagged TAZ or TAZ4SA, cell lysates were immunoprecipitated with anti‐Flag antibody, and the bound components were analysed in the presence of MG132, a proteasome inhibitor. As shown in Figure 3A, Flag‐TAZ interacted with Atrogin‐1 and MuRF1, and the interaction was further enhanced in the presence of dexamethasone [F‐TAZ; DEX(−) vs. (+): Atrogin‐1 (1.68‐fold, *p* = 0.025), MuRF1 (1.60‐fold, *p* = 0.019)]. However, the interaction between Flag‐TAZ4SA and Atrogin‐1/MuRF1 was found to be reduced in comparison to the interaction between Flag‐TAZ and Atrogin‐1/MuRF1 [DEX(−); F‐TAZ vs. F‐TAZ4SA: Atrogin‐1 (0.44‐fold, *p* = 0.046), MuRF1 (0.44‐fold, *p* = 0.019)]. The interaction between Flag‐TAZ4SA and Atrogin‐1/MuRF1 was not discernible even in the presence of dexamethasone [F‐TAZ4SA; DEX(−) vs. (+): Atrogin‐1 (1.05‐fold, *non‐significant*), MuRF1 (0.96‐fold, *non‐significant*)] (Figure 3A). The results indicate that dexamethasone‐induced Atrogin‐1/MuRF1 facilitates the degradation of TAZ.

To further investigate the degradation of TAZ by Atrogin‐1 and MuRF1, Atrogin‐1 or MuRF1 was depleted using the CRISPR‐Cas9 system in C2C12 cells. The cells depleted of Atrogin‐1 or MuRF1 were treated with dexamethasone, and the levels of TAZ were analysed. As shown in Figure 3B, Atrogin‐1 levels were diminished in Atrogin‐1 KO cells relative to control cells (0.51‐fold, *p* = 0.025). The Atrogin‐1 KO cells exhibited a significant increase in TAZ levels (2.05‐fold, *p* = 0.007). Similarly, as shown in Figure 3C, MuRF1 levels were diminished in MuRF1 KO cells relative to control cells (0.53‐fold, *p* = 0.006). Additionally, TAZ levels were increased in MuRF 1 KO cells (2.33‐fold, *p* = 0.013). These results suggest that TAZ is a target protein for Atrogin‐1 and MuRF1 in dexamethasone‐induced muscle atrophy.

### 3.4 TAZ Stimulates mTOR Signalling

**FIGURE 4:**
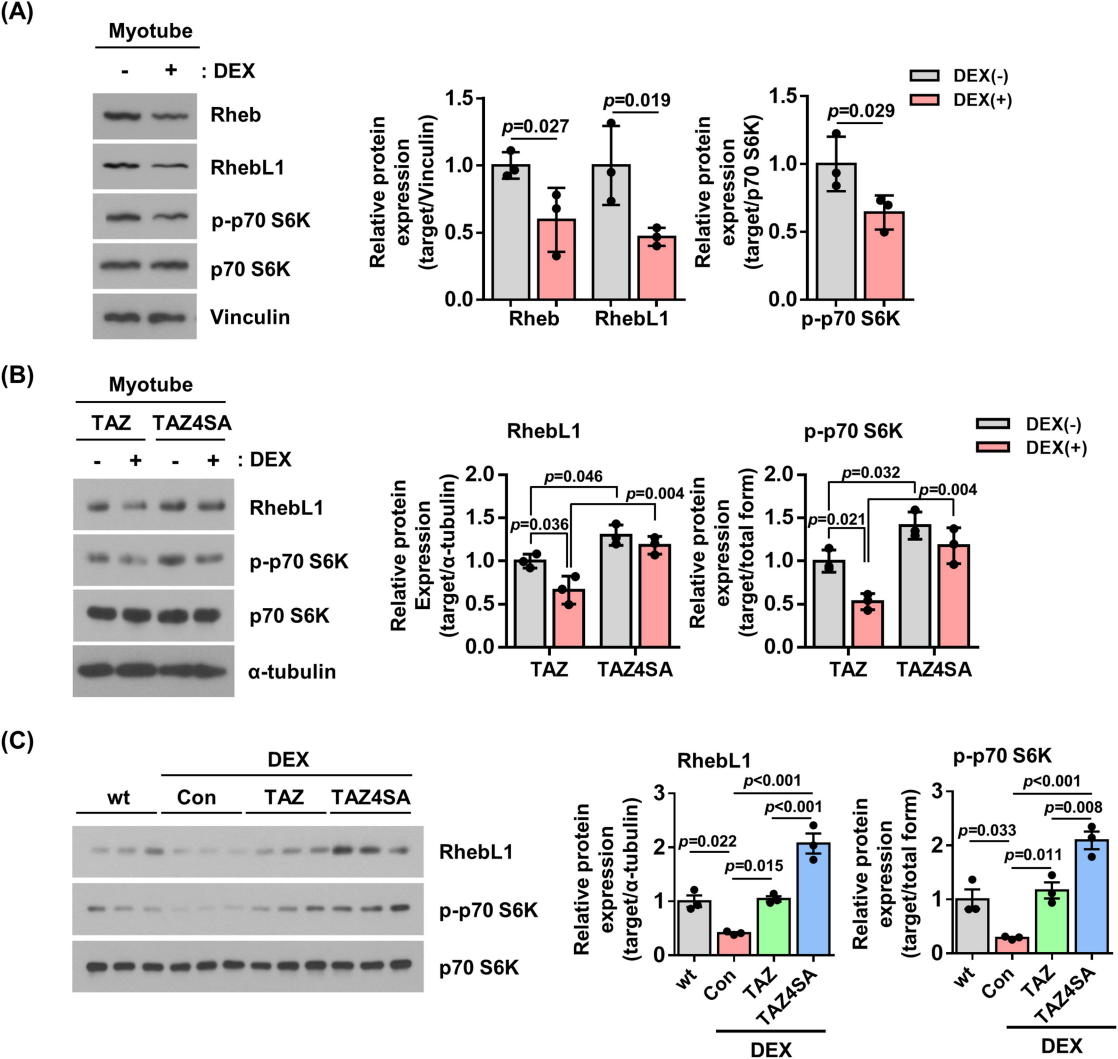
TAZ stimulates mTOR signalling. (A) Left: C2C12 myotubes were treated with either a vehicle [DEX(−)] or dexamethasone [DEX(+)]. The levels of Rheb, RhebL1, phospho‐p70 S6K (p‐p70 S6K), p70 S6K and vinculin were analysed by immunoblotting. Right: The protein levels depicted in the left panel were quantified (*n* = 3). (B) Left: TAZ‐ and TAZ4SA‐overexpressing C2C12 myotubes were treated with either a vehicle [DEX(−)] or dexamethasone [DEX(+)]. The levels of RhebL1, p‐p70 S6K, p70 S6K and α‐tubulin were analysed by immunoblotting. Right: The protein levels shown in the left panel were quantified (*n* = 3). (C) Left: The control (Con), TAZ and TAZ4SA AAV viruses were introduced into the GA muscle of mice. Following dexamethasone administration, gastrocnemius muscle was isolated, and the levels of RhebL1, p‐p70 S6K and p70 S6K were analysed by immunoblotting. Right: The protein levels of the left panel were quantified (*n* = 3). α‐Tubulin in Figure 2F was used for the quantification of RhebL1. The data are presented as the mean ± SD for Panels (A) and (B) and as the mean ± SEM for Panel (C). Statistical analysis was performed using appropriate tests: Panel (A) was analysed using one‐tailed *t*‐test, Panel (B) was analysed using two‐way ANOVA followed by Holm–Sidak's multiple comparisons test, and Panel (C) was analysed using one‐way ANOVA followed by Tukey's multiple comparisons test. Exact *p* values are provided in the graph, and *non‐significant* results are not explicitly indicated.

mTOR signalling plays a key role in protein synthesis [28, 29]. It was observed that TAZ stimulates mTOR signalling through the induction of Rheb/RhebL1 [23]. We next investigated whether TAZ‐mediated mTOR signalling is involved in dexamethasone‐induced muscle atrophy. As shown in Figure 4A, dexamethasone resulted in a reduction in the levels of Rheb, RhebL1 and phosphorylated p70 S6K [Rheb (0.59‐fold, *p* = 0.027), RhebL1 (0.46‐fold, *p* = 0.019), p‐p70 S6K (0.64‐fold, *p* = 0.029)]. Moreover, the protein levels were compared in C2C12 cells overexpressing TAZ or TAZ4SA. As shown in Figure 4B, TAZ‐overexpressing cells show a reduction in RhebL1 and phosphorylated p70 S6K following dexamethasone treatment [TAZ; DEX(−) vs. DEX(+): RhebL1 (0.66‐fold, *p* = 0.036), p‐p70 S6K (0.53‐fold, *p* = 0.021)]. Prior to dexamethasone treatment, TAZ4SA‐overexpressing cells exhibited higher levels of RhebL1 and p‐p70 S6K compared to TAZ‐overexpressing cells [DEX(−); TAZ vs. TAZ4SA: RhebL1 (1.30‐fold, *p* = 0.046), p‐p70 S6K (1.41‐fold, *p* = 0.032)]. However, the decrease was not as pronounced in TAZ4SA‐overexpressing cells [TAZ4SA; DEX(−) vs. DEX(+): RhebL1 (0.91‐fold, *non‐significant*), p‐p70 S6K (0.84‐fold, *non‐significant*)]. Furthermore, the effect of TAZ4SA in vivo was investigated. After introducing TAZ‐ or TAZ4SA‐overexpressing adeno‐associated virus into the mouse muscle, dexamethasone treatment was performed, and the levels of RhebL1 and phosphorylated p70 S6K were analysed. As shown in Figure 4C, the TAZ4SA mutant demonstrated markedly elevated levels of RhebL1 and phosphorylated p70 S6K in comparison to TAZ [DEX; TAZ vs. TAZ4SA: RhebL1 (1.98‐fold, *p <* 0.001), p‐p70 S6K (1.80‐fold, *p* = 0.008)]. These results suggest that TAZ plays an important role in the prevention of muscle atrophy by activating the mTOR pathway.

### 3.5 Ginsenoside Rb3 Increases TAZ Levels and Inhibits Muscle Atrophy

**FIGURE 5:**
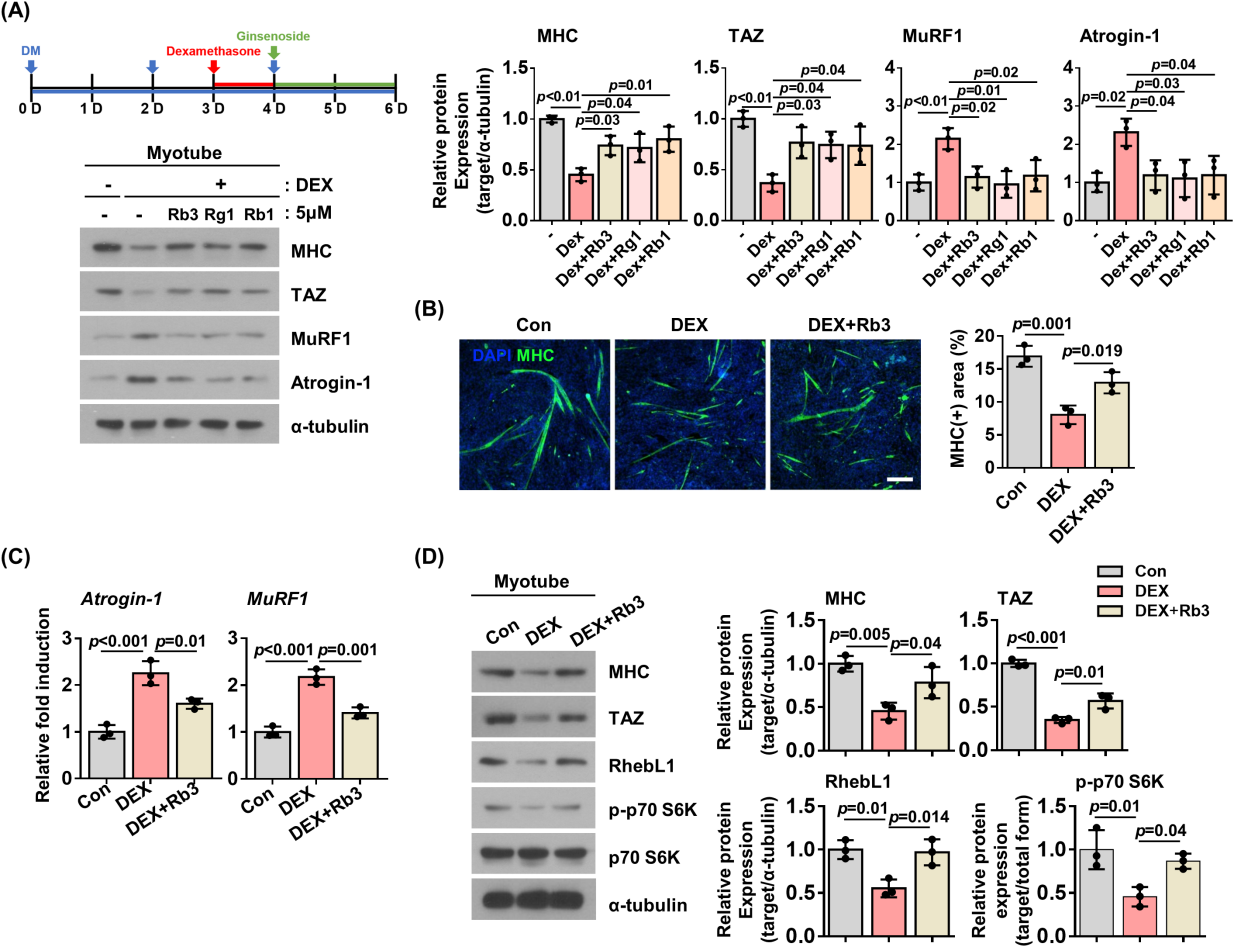
Ginsenoside Rb3 inhibits dexamethasone‐induced TAZ depletion. (A) Left: Schematic representation of the experimental design for the ginsenoside treatment in C2C12 myotubes. DM refers to differentiation media. Ginsenosides Rb3, Rg1 and Rb1 were administered to C2C12 myotubes that had been treated with dexamethasone. The levels of MHC, TAZ, MuRF1, Atrogin‐1 and α‐tubulin levels were analysed by immunoblotting. α‐tubulin was used as a loading control. Right: The levels of MHC, TAZ, MuRF1 and Atrogin‐1 were quantified (*n* = 3). (B) Left: The levels of MHC was analysed by immunofluorescence staining in C2C12 myotubes treated with a vehicle (Con), dexamethasone (DEX) and dexamethasone plus ginsenoside Rb3 (DEX + Rb3). Scale bar, 100 μm. Right: The MHC‐positive area was quantified from the immunofluorescence staining (*n* = 3). (C) The transcript levels of *Atrogin‐1* and *MuRF1* were analysed by qRT‐PCR in Panel (B) (*n* = 3). (D) Left: The levels of MHC, TAZ, RhebL1, p‐p70 S6K, p70 S6K and α‐tubulin were analysed by immunoblotting in Panel (B). α‐tubulin was used as a loading control. Right: The protein levels of the left panel were quantified (*n* = 3). All data are presented as the mean ± SD. Statistical analysis for all figures was performed using one‐way ANOVA followed by Tukey's multiple comparisons test. Exact *p* values are provided in the graph, and *non‐significant* results are not explicitly indicated.

Ginsenosides, the principal active constituents of *Panax ginseng* , have been shown to be able to alleviate muscle atrophy [30, 31, 32]. To identify ginsenosides that inhibit muscle atrophy via TAZ, ginsenosides Rb3, Rg1 and Rb1 were administered to dexamethasone‐treated C2C12 myotubes. As shown in Figure 5A, ginsenosides Rb3, Rg1 and Rb1 inhibited muscle atrophy, as evidenced by increased MHC levels compared to vehicle control [(−) vs. DEX; MHC: Rb3 (1.64‐fold, *p* = 0.034), Rg1 (1.58‐fold, *p* = 0.043), Rb1 (1.77‐fold, *p* = 0.011)]. Additionally, TAZ levels are increased in ginsenoside‐treated myotubes relative to vehicle‐treated myotubes [(−) vs. DEX; TAZ: Rb3 (2.08‐fold, *p* = 0.029), Rg1 (2.01‐fold, *p* = 0.039), Rb1 (1.99‐fold, *p* = 0.043)] (Figure 5A). Subsequent analysis was evaluated with ginsenoside Rb3. The increased MHC levels resulting from ginsenoside Rb3 was further verified by immunostaining analysis (DEX vs DEX + Rb3: 1.61 fold, *p* = 0.019) (Figure 5B). Ginsenoside Rb3 inhibited dexamethasone‐induced Atrogin‐1 and MuRF1 transcription [DEX vs. DEX + Rb3: *Atrogin‐1* (0.71‐fold, *p* = 0.011), *MuRF1* (0.65‐fold, *p* = 0.001)] (Figure 5C). Furthermore, as shown in Figure 5D, dexamethasone administration resulted in a reduction in RhebL1 and phosphorylated p70 S6K levels [Con vs. DEX: RhebL1 (0.55‐fold, *p* = 0.010), p‐p70 S6K (0.46‐fold, *p* = 0.012)]. However, the decrease was not observed after ginsenoside Rb3 treatment [Con vs. DEX + Rb3: RhebL1 (0.97‐fold, *non‐significant*), p‐p70 S6K (0.87‐fold, *non‐significant*)].

**FIGURE 6:**
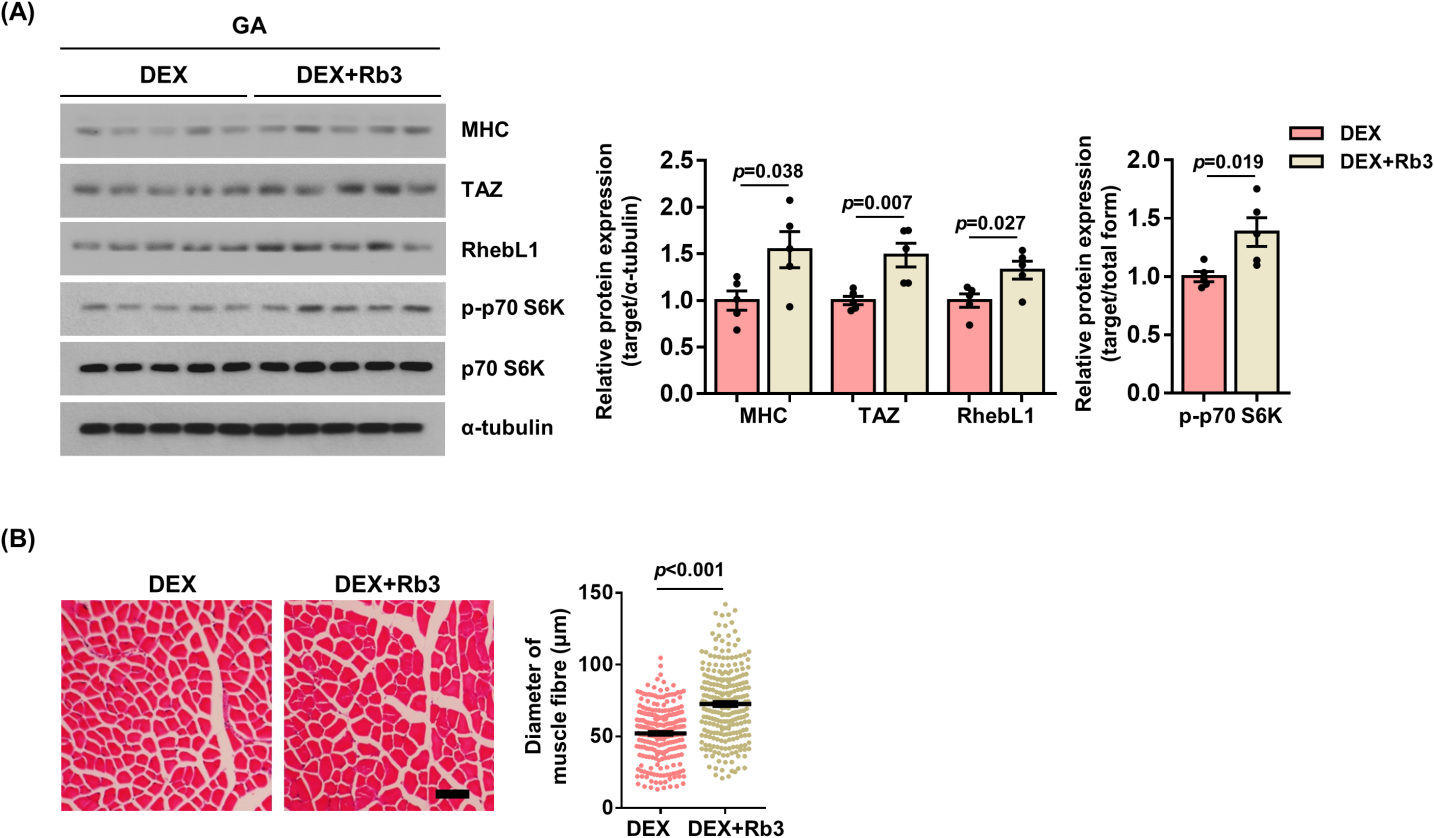
Ginsenoside Rb3 inhibits dexamethasone‐induced muscle atrophy through the induction of TAZ. (A) During dexamethasone‐induced muscle atrophy, ginsenoside Rb3 was administered orally at a dose of 10 mg/kg. Subsequently, the GA muscles from the mice treated with dexamethasone‐only (DEX) and dexamethasone plus ginsenoside Rb3 (DEX + Rb3) were isolated and subjected to analysis. The levels of MHC, TAZ, RhebL1, p‐p70 S6K, p70 S6K and α‐tubulin were analysed by immunoblotting. α‐Tubulin was used as a loading control. Right: The protein levels shown in the left panel were quantified (*n* = 5). (B) Left: H&E staining of GA muscles in Panel (A). Scale bar, 50 μm. Right: Muscle fibre diameters were quantified using ImageJ software. Each dot on the graph represents the diameter of an individual muscle fibre, and the total number of fibres measured was over 250 per group. Histological analysis was performed on muscle samples from five mice per group. All data are presented as the mean ± SEM. Statistical analysis for all figures was performed using two‐tailed *t*‐test. Exact *p* values are provided in the graph, and *non‐significant* results are not explicitly indicated.

To investigate the effect of ginsenoside Rb3 in vivo, Rb3 was administered to dexamethasone‐treated mice. As shown in Figure 6A, the administration of ginsenoside Rb3 resulted in increased levels of MHC and TAZ compared to vehicle control [DEX vs. DEX + Rb3: MHC (1.54‐fold, *p* = 0.038), TAZ (1.49‐fold, *p* = 0.007)]. Additionally, the levels of RhebL1 and phosphorylated p70 S6K were increased in ginsenoside Rb3‐treated mice relative to control mice [DEX vs. DEX + Rb3: RhebL1 (1.33‐fold, *p* = 0.027), p‐p70 S6K (1.38‐fold, *p* = 0.019)] (Figure 6A). In addition, the diameter of muscle fibre was also observed to be larger in ginsenoside Rb3‐treated mice compared to control mice (1.39‐fold, *p* < 0.001) (Figure 6B). These results suggest that ginsenoside Rb3 may exert an inhibitory effect on dexamethasone‐induced muscle atrophy through the induction of TAZ and the activation of the mTOR signalling.

## 4 Discussion

Chronic treatment with dexamethasone has been demonstrated to have adverse effects, including muscle atrophy. Dexamethasone has been shown to reduce muscle mass and stimulate muscle protein degradation. It has been demonstrated that dexamethasone stimulates the transcription of muscle atrophic genes, including Atrogin‐1 and MuRF1, while also inhibiting hypertrophic signalling pathways, such as those involving mTOR. The present study demonstrates that TAZ is a downstream effector of dexamethasone‐induced muscle atrophy. Dexamethasone was observed to reduce TAZ levels with a reduction in MHC levels in myotubes (Figure 1B) and muscle tissue (Figure 1E). In contrast, TAZ mutants resistant to degradation were observed to inhibit the reduction in muscle fibre diameter induced by dexamethasone (Figure 2E) and the reduction in MHC levels (Figure 2F). Further study demonstrated that dexamethasone‐induced Atrogin‐1 and MuRF1 interact with TAZ, thereby facilitating its degradation (Figure 3A). The depletion of Atrogin‐1 or MuRF1 was observed to inhibit dexamethasone‐induced TAZ degradation (Figure 3B,C). These results suggest that TAZ plays an important role in dexamethasone‐induced muscle atrophy and that maintaining TAZ levels may represent a viable therapeutic strategy for alleviating muscle atrophy.

It has been demonstrated that exercise can counteract the deleterious effects of the ageing process, including the reduction of muscle mass and the decline in mitochondrial respiration [33]. It has been previously demonstrated that TAZ is induced following endurance exercise and that this stimulates mitochondrial biogenesis via the mTOR pathway [23]. Furthermore, our research has demonstrated that TAZ induction by exercise plays a crucial role in facilitating muscle adaptation to exercise [24]. It has been shown that exercise can mitigate dexamethasone‐induced muscle atrophy [34, 35, 36]. However, the precise mechanism by which exercise training attenuates muscle atrophy remains to be elucidated. In this study, we observed that dexamethasone resulted in a reduction in TAZ levels and a downregulation of mTOR signalling via the TAZ‐Rheb/RhebL1 axis (Figure 4B,C). Consequently, our results suggest that inducing TAZ levels through exercise training may be beneficial in maintaining muscle mass and improving mitochondrial function in dexamethasone‐induced muscle atrophy.

In our previous study [23], we reported that muscle‐specific TAZ knockout mice had comparable muscle mass to wild‐type mice, suggesting that TAZ may not be essential for maintaining muscle mass under normal physiological conditions. However, in the cardiotoxin‐induced muscle injury model, TAZ levels increase significantly, and it stimulates muscle regeneration [21]. In a dexamethasone‐induced muscle atrophy model, significantly reduced TAZ levels are observed, and it exacerbates muscle atrophy. These findings indicate that TAZ is not required for the maintenance of muscle mass under normal conditions but plays a critical role in muscle regeneration under pathological conditions, suggesting that the role of TAZ in muscle homeostasis is context dependent.

A number of studies have shown that ginsenosides including Rh1, Rg2 and Rg3 have the ability to alleviate dexamethasone‐induced muscle atrophy through the modulation of the AKT/mTOR signalling pathway [30, 31, 32]. The present study investigated the effect of ginsenosides Rb3, Rg1 and Rb1 on dexamethasone‐induced muscle atrophy. The administration of these ginsenosides resulted in an increase in TAZ levels and a reduction in dexamethasone‐induced muscle atrophy in C2C12 myotubes (Figure 5A). Furthermore, ginsenoside Rb3 was observed to inhibit dexamethasone‐induced muscle atrophy in vivo (Figure 6). Ginsenoside Rb3 has been observed to increase TAZ levels through transcription, which in turn induces RhebL1 to stimulate mTOR signalling pathway. The results suggest that TAZ may play a role in mediating the effect of ginsenoside in attenuating dexamethasone‐induced muscle atrophy.

In conclusion, the findings of this study demonstrate that TAZ is an effector protein for dexamethasone‐induced muscle atrophy and that it inhibits the process of muscle atrophy. These results suggest that the maintenance of TAZ levels is crucial for the prevention of dexamethasone‐induced muscle atrophy.

## Author Contributions

K.M.K. performed the experiments, analysed the data and wrote the manuscript. H.T.O., Y.D., G.D.Y., W.H., J.P., H.Y., S.J.Y. and M.R.B. prepared the reagents used for the experiments, set up the experimental systems and analysed the experimental data. E.S.H. and J.H.H. led the project, interpreted the data and wrote the manuscript.

## Conflicts of Interest

The authors declare no conflicts of interest..

## Data Availability

This study does not include any data that need to be deposited in external repositories.
